# Is There a Global Bioethics? End-of-Life in Thailand and the Case for Local Difference

**DOI:** 10.1371/journal.pmed.0030439

**Published:** 2006-10-24

**Authors:** Scott Stonington, Pinit Ratanakul

## Abstract

Western bioethics is insufficient, say the authors, to solve the problems created by the adoption of allopathic medicine in non-Western contexts.

Over the past decade, several scholars have advocated for international standards in medical ethics and human rights [1–3]. Others have countered that such standards risk ignoring important cultural differences in the way people conceptualize medical decision-making [4–8]. Within this debate hangs a question for international bioethics: as developing countries build allopathic medical systems, what should their bioethics be? In this essay, we explore possible answers to this question, ultimately arguing that Western bioethics is insufficient to solve the problems that arise in the practice of allopathic medicine in non-Western contexts.

As an example, we discuss recent conflicts over the use of mechanical ventilators in Thailand. Thailand is a center of cutting-edge allopathic medical care in Asia. It has a universal health-care system, which provides many Thais with access to mechanical ventilation. So many Thais are placed on mechanical ventilators at the end of life that it has become one of the largest drains on Thailand's universal health-care system [9]. Furthermore, the use of ventilators has become a source of vehement national debate, mostly as a result of several prominent political figures who received overly aggressive medical care at the end of life [10,11]. As in Western hospitals, the ascension of mechanical ventilation has introduced a host of difficult ethical dilemmas for doctors, families, and patients [12,13]. How will Thais go about solving these dilemmas? On which principles of bioethics will they rely?

**Figure:**
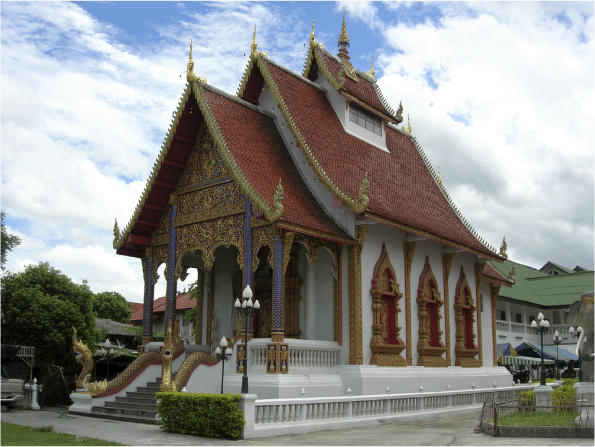
Most hospitals in Thailand have temples nearby where patients and families can grapple with the karmic landscape of illness and medicine (Photo: Scott Stonington)

To answer these questions, we start with a case that illustrates a common ethical dilemma about withdrawal of mechanical ventilation in Thai intensive care units. We then explore some concepts from Western bioethics to see if they help resolve this dilemma. Finally, we explain some of the local ethics behind the case and discuss the concept of a Thai bioethics to address the use of ventilators in Thailand.

## A Case Scenario

The following fictional case is based on 30 ethnographic interviews and two months of participant-observation fieldwork by one of us (SS) in 2005. The case contains themes that arose frequently during this research.

Gaew, a 39-year-old Thai construction worker, falls from a scaffold and hits his head on the pavement. He is unconscious by the time he arrives at one of Bangkok's cutting-edge emergency rooms. He is intubated and placed in the intensive care unit. Gaew's physician, Dr. Nok, informs Gaew's brother, Lek, that Gaew has little chance of recovery due to his lack of brain activity.

Lek does not know what to do—he wants to give his brother the best care possible, but he knows his brother is suffering. He would like to remove Gaew's ventilator. Dr. Nok replies that this is impossible because it is unethical to remove ventilators. Very few physicians in Thailand withdraw ventilators from patients [10]. They have a complex array of reasons for declining to withdraw ventilator support, including their medical training, fear of litigation, and belief in the sanctity of life.

As with most Thai physicians, Dr. Nok's refusal to withdraw the ventilator is explicitly Buddhist. The first precept of Buddhism forbids killing. Other Buddhist doctrines teach that the last part of the body to die is the breath. For a Thai Buddhist physician, pulling out a patient's ventilator may feel like pulling out the patient's soul. If Dr. Nok withdraws Gaew's ventilator, she will necessarily have “ill-will” or “repugnance” in her mind [14,15]. In Buddhist terms, Dr. Nok's own karma is at stake. Karma is a moral law, central to lay Thai Buddhism, which describes chains of cause and effect that result from individual behavior. Actions generate either merit or demerit, and the balance of these two currencies determines one's spiritual future [10,15,16]. If Dr. Nok's mind contains ill-will or repugnance, she will accrue demerit, which will negatively affect her in this and future lifetimes.

Neither Lek nor Dr. Nok ask what Gaew would have wanted in his current situation. They do not ponder this question because in lay Thai Buddhism, the self is seen as different from moment to moment—so Gaew is not the same person now as he was ten days ago. To Dr. Nok and Lek, an advance directive seems ludicrous. How could a person know what he would want years later, in a different state of consciousness [10]?

Dr. Nok is ready with a strategy for circumventing their dilemma. She tells Lek that together they must help Gaew “let go.” She explains that it is Gaew's mental attachments that are keeping him alive and suffering on the ventilator. When Dr. Nok says “attachments,” she uses the Thai word for “knot of problems” (bpom bpan ha), implying a gnarled set of worries tangling Gaew's mind and keeping him from achieving mental clarity and letting go of life. She asks Lek what Gaew might be worried about. Lek replies that Gaew wanted to ordain as a monk before dying. Although they cannot know what is in Gaew's mind in his new state of consciousness, this is a possible element in his “knot.”

Dr. Nok suggests that Lek go to Bangkok and ordain as a monk for several days in Gaew's stead, then return to tell Gaew what he has done. She explains that even though Gaew has little brain activity, when all of the senses subside, the spirit may still take in sound [15]. She hopes that when Gaew hears about his brother's ordination, he may let go and die with the ventilator still attached and running. This way, she and Lek can relieve Gaew's suffering without compromising their karma.

## How Would Western Bioethics Handle this Case?

There has been a recent fervor of discussion in many Western medical schools about culture and bioethics [8]. Medical students and physicians are being trained in “cultural competence” to help them handle a culturally diverse society. This training usually focuses on prototypic cases meant to exemplify particular cultural or ethnic groups. In general, it is assumed that the principles of Western bioethics—autonomy, beneficence, non-maleficence, truth-telling, and justice—are universal. Different cultures are seen as emphasizing these principles differently, rather than as operating on unique principles of their own.

A classic example, taught in many United States medical schools, is the story of the “Asian” elder who comes into the hospital, and whose son says “please, do not tell my father that he has cancer.” Most Western physicians would analyze this situation as follows: the son believes that knowing about the illness will hurt his father; the son values beneficence (doing what is best for the patient) over autonomy (the patient's prerogative to make decisions for himself) and thus wants to conceal the illness from his father. In this analysis, the principles of bioethics are held to be universal—the son's culture simply makes him value these principles in a unique proportion.

**Figure:**
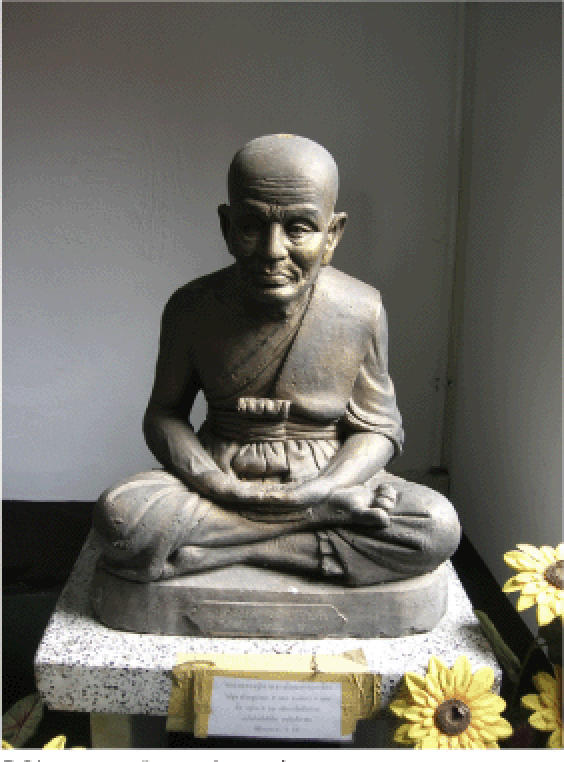
Statues of monks, like this figure at Wat U Mong Klang Wiang, are common sites for Thai Buddhists' offerings (Photo: Scott Stonington)

This approach proves unhelpful in understanding Gaew's case. Dr. Nok's refusal to remove the ventilator is not based on Gaew's wishes; it is not based on what is best for Gaew; and it is not about what is most truthful, or what is best for Thais as a whole. None of these fundamental principles of Western bioethics—autonomy, beneficence, non-maleficence, truth-telling, or justice—sufficiently explain Lek and Dr. Nok's dilemma. Even though the hospital taking care of Gaew is a center of allopathic medicine—a form of medicine grown out of the West—it is nonetheless a zone governed at least partially by non-Western bioethical principles.

A tool central to the practice of bioethics in Western hospitals is delineating between different kinds of dilemmas. The most widely read textbook of bioethics in the West, by Beauchamp and Childress, distinguishes between at least three kinds of dilemmas: (1) *ethical dilemmas*, where two ethical principles dictate opposite actions; (2) *self-interest dilemmas*, where the decision-maker's own self-interest conflicts with a decision dictated by an ethical principle; and (3) *practical dilemmas*, where something logistical prevents an ethical decision from being enacted [17]. Making these distinctions is often the first task that a physician must complete during an ethics consult. One must separate the entangled needs of doctors and family members from the ethical principles that determine how to treat a patient.

So what kind of dilemma are Lek and Dr. Nok confronting? Are the principles governing their behavior ethical, practical, or self-interested? Take, for example, Dr. Nok's reason for not withdrawing the ventilator: to do so would be revoking a patient's life. At first, this sounds like an ethical principle, a kind of non-maleficence. But on closer inspection, the principle beneath her action diverges significantly from non-maleficence. In a Buddhist framework, killing is ethically wrong because it defiles the mind of the killer. Even if Dr. Nok thinks that withdrawing the ventilator is the most compassionate thing for Gaew, it would be spiritually disadvantageous for her. As one Thai physician explained, “it may be the best thing for the patient [to withdraw the ventilator], but how could you find someone who would do it?” A Thai physician would not want to take the risk of acquiring spiritual demerit.

It would then be tempting to say that Dr. Nok's situation represents a self-interest dilemma. An ethical decision—compassionately relieving suffering by removing the ventilator—is in conflict with Dr. Nok's concern for her own spiritual fate. But this interpretation also breaks down because the precise thing that would generate demerit for Dr. Nok is ill-will toward Gaew. In a Buddhist ethical framework, it is impossible to withdraw a ventilator with beneficent intent. In Dr. Nok's case, self-interest and ethical duty are so intertwined as to be indistinguishable. The distinction made between self-interest and ethical dilemmas collapses. The first task of a Western ethicist—to determine the type of dilemma at work—proves an impasse in Gaew's case.

The fact that a Western bioethical approach fails in Gaew's case may be an indication of the limitations of the “one-size-fits-all” bioethics used in Western hospitals as much as it is an illustration of local differences in ethical reasoning (Damien Keown, personal correspondence). Western bioethics is a young discipline, and draws on only a minority of the rich history of Western ethical philosophy [18]. Nonetheless, the conceptual tools of Western bioethics dominate policy, law, bureaucracy, and physician decision-making in Western hospitals. These concepts are beginning to have weight in policy-making in Thailand [19]. Gaew's case makes it clear that one must examine local ethical concepts before uncritically importing Western bioethical tools.

## Does Thailand Need a Thai Bioethics?

Dr. Nok's solution to Gaew's end-of-life is instructive as an introduction to what a Thai bioethics might look like. Dr. Nok and Lek cannot remove Gaew's ventilator, and yet their compassion and duty demand that they relieve his suffering. They circumvent this dilemma by helping Gaew to let go of his life peacefully. This strategy has a positive effect on the karmic fate of everyone involved. They relieve Gaew's suffering. Lek acquires merit by ordaining as a monk.

These decisions are based on the logic of karmic morality. They also illustrate the Buddhist principle of interdependence. Interdependence means that doctors, patients and relatives must think about the emotions and interests of all parties involved in a medical decision. This is in contrast to the Western concept of autonomy, which allows a patient to make decisions without consideration of the feelings and responsibilities of other people concerned. Dr. Nok's solution to Gaew's end-of-life is not just for Gaew, it is also for herself and for Lek. It is an ethics of compassion that must relieve the suffering of all people concerned.

One of us (PR), as a member of a team of Thai scholars, has worked for the last ten years to develop an applied ethics using principles such as karma, compassion, and interdependence [20–23]. In the West, the main purpose of a country-wide policy is to resolve conflicts between individuals over medical decisions. However, because the concept of interdependence is so central for most Thais, Thailand's bioethical policies may differ dramatically from those found in the West.

## Conclusion

The purpose of this exploration has been to illustrate the need for Thailand and other countries to develop bioethical systems using local concepts. It would be a mistake, however, to leave our analysis of Thai bioethics without considering the term “Thai.” This has long been a problem with writings on “Asian values” or “Asian thinking.”

In this article, we have emphasized Buddhism as a major ethical system, but it is one of many such systems engaged in decisions about the end-of-life in Thailand. Buddhist monasteries, lay Buddhist organizations, advocates of medical technology, public health officials, and lobbyists for the booming medical tourism industry are all engaged in vehement debate over what should guide Thailand in making medical decisions [10,11]. As with other countries, Thailand is not a place with a single ethics. In the same way that one cannot import concepts from the West to solve dilemmas in Thailand, one cannot haphazardly select a view within Thailand and label it as “Thai.”

Nonetheless, there is an urgent need for solutions to the “ventilator problem”—both to patch the failing universal health-care system and to help Thais make difficult decisions about intervention at the end-of-life. Thailand is just beginning the long process of integrating its multitude of local voices and concepts into nationwide ethical standards. This new Thai ethics promises to be much more effective at solving Thailand's ethical problems than tools imported uncritically from the West.
